# Supplementary material for: Thicker Retinal Nerve Fiber Layer with Age among Schoolchildren: The Hong Kong Children Eye Study
Source: Diagnostics (Basel). 2022 Feb 15;12(2):500. doi: 10.3390/diagnostics12020500 (PMC8870939; doi:10.3390/diagnostics12020500)
Supplement: Supplementary file 1 [file diagnostics-12-00500-s001.zip › diagnostics-1587898-supplementary.pdf]

diagnostics

Supplementary Table S1. Global RNFL Thickness by Different Age in Myopia, Emmetropia and Hyperopia Groups.

	Myopia		Emmetropia		Hyperopia	
**Age Group, yrs**	**Mean**	**SD**	**Mean**	**SD**	**Mean**	**SD**
6	100.56	0.67	105.12	0.48	107.92	0.32
7	102.24	0.47	105.91	0.46	108.43	0.32
8	103.58	0.41	106.66	0.44	109.37	0.41
**Overall *p* Values**	0.001		0.028		0.023	

Mean and SD was calculated with both eye data. SD = Standard Deviation; Significant was set at 0.05. *p* values were generated by generalized estimating equation models with sex, AL, BMI, IOP, CCT and BP and baseline SE adjustment for SE comparisons.
